# Regulation of Ack1 localization and activity by the amino-terminal SAM domain

**DOI:** 10.1186/1471-2091-11-42

**Published:** 2010-10-27

**Authors:** Victoria Prieto-Echagüe, Azad Gucwa, Deborah A Brown, W Todd Miller

**Affiliations:** 1 Department of Physiology and Biophysics, Basic Science Tower T5, School of Medicine, Stony Brook University, Stony Brook, NY 11794-8661, USA; 2 Department of Biochemistry and Cell Biology, Nicolls Rd., Stony Brook University, Stony Brook, NY 11794-5215, USA

## Abstract

**Background:**

The mechanisms that regulate the activity of the nonreceptor tyrosine kinase Ack1 (activated Cdc42-associated kinase) are poorly understood. The amino-terminal region of Ack1 is predicted to contain a sterile alpha motif (SAM) domain. SAM domains share a common fold and mediate protein-protein interactions in a wide variety of proteins. Here, we addressed the importance of the Ack1 SAM domain in kinase activity.

**Results:**

We used immunofluorescence and Western blotting to show that Ack1 deletion mutants lacking the N-terminus displayed significantly reduced autophosphorylation in cells. A minimal construct comprising the N-terminus and kinase domain (NKD) was autophosphorylated, while the kinase domain alone (KD) was not. When expressed in mammalian cells, NKD localized to the plasma membrane, while KD showed a more diffuse cytosolic localization. Co-immunoprecipitation experiments showed a stronger interaction between full length Ack1 and NKD than between full length Ack1 and KD, indicating that the N-terminus was important for Ack1 dimerization. Increasing the local concentration of purified Ack1 kinase domain at the surface of lipid vesicles stimulated autophosphorylation and catalytic activity, consistent with a requirement for dimerization and trans-phosphorylation for activity.

**Conclusions:**

Collectively, the data suggest that the N-terminus of Ack1 promotes membrane localization and dimerization to allow for autophosphorylation.

## Background

**Figure 1:**
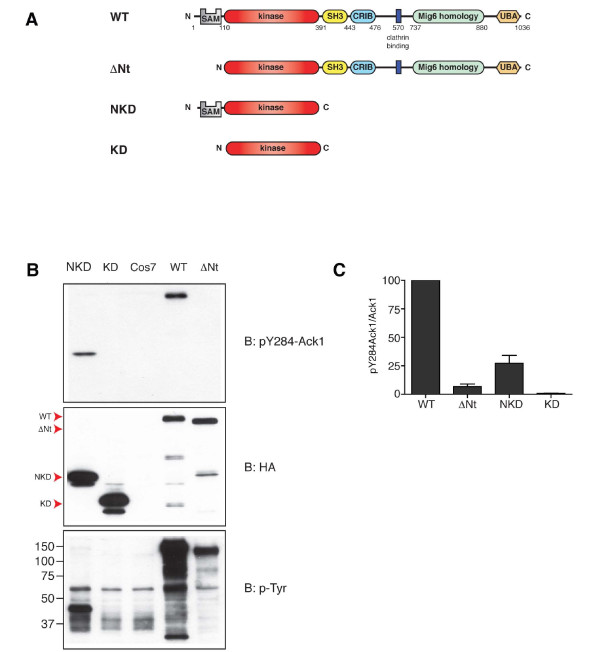
**The N-terminus of Ack1 is required for autophosphorylation in cells**. A, constructs used in this study: WT, full length Ack1 (accession # Q07912); ΔNt, deletion of the N-terminal 110 amino acids; NKD, amino terminus plus kinase domain (residues 1-381); KD, kinase domain alone (residues 111-381). B, Western blot analysis. Lysates from Cos7 cells expressing wild type or truncated forms of Ack1 were probed with anti- phospho Ack1 (pY284), anti-HA, and anti-phosphotyrosine antibodies as indicated. The figure is representative of five experiments. C, densitometry readings were taken from all five experiments and the average was plotted in a bar graph. Error bars indicate standard error.

Ack1 is a 120 kDa non-receptor tyrosine kinase (NRTK) with the domain arrangement shown in Figure 1A. From N- to C-terminus, Ack1 contains a sterile alpha motif (SAM) domain [1], a kinase domain, an SH3 domain, a Cdc42-binding domain (CRIB), a clathrin-binding motif [2], a region homologous to Mig6 [3], and a ubiquitin binding domain [4]. The C-terminal portion of Ack1 also contains several proline-rich sequences that serve as protein-protein interaction motifs [5-8]. Members of the Ack family include human Ack1/Tnk2 [9], Tnk1/Kos1 in human and mouse [10,11], bovine Ack2 [12], DACK and DPR2 in *Drosophila melanogaster *[13] and Ark-1 in *Caenorhabditis elegans *[14]. The isoforms share the overall domain arrangement of Ack1 (i.e. a kinase domain followed by an SH3 domain and Pro-rich sequences), but differ in some details. For instance, Ack2 has a shorter N-terminal region and a shorter C-terminal portion [12]. The isoforms Tnk1, Kos1 and DACK do not have a CRIB domain and are shorter than Ack1 [10,13,15].

Several proteins have been shown to interact with Ack1. GTP-bound Cdc42 binds to the CRIB domain and activates Ack1 [9]. Ligands for the proline-rich region of Ack1 include the SH3 domains of Hck [8], Grb2 [6], SNX9 [6,7], and the WW domain of the E3 ubiquitin ligase Nedd4-2 [5]. Upon activation by Cdc42, Ack1 phosphorylates the guanine exchange factor Dbl [16,17] and the adapter protein p130Cas [18]. Although the physiological roles of Ack1 are still unclear, it has been implicated in several signaling pathways. Ack1 is phosphorylated and activated in response to multiple growth factors [1,6] and integrin-mediated signals [18,19]. Ack1 participates in a signaling complex with EGFR [4]. In addition, due to its interaction with proteins such as ubiquitin [4], clathrin heavy chain [2,20] and the sorting nexin SH3PX1 [21], Ack1 is thought to play a role in the regulation of EGFR stability and vesicle dynamics.

SAM (sterile-alpha motif) domains are modular domains of ~70 amino acids that were originally identified and named due to their essential role in sexual differentiation in yeasts. In spite of their common five-helical structure, SAM domains do not exhibit significant sequence homology [22]. This sequence diversity reflects a large diversity of function. SAM domains have been found in nearly 1000 proteins that include a diverse array of signaling molecules such as the transcriptional repressors TEL [23] and Polycomb [24], diacyglycerol kinase δ1 (DGKδ1) [25], and Eph receptor tyrosine kinases [26,27].

Structural studies of the SAM domains from the EphA4 receptor [26] and EphB2 receptor [27] showed that the helical arrangement creates two interfaces, suggesting a mechanism for SAM domain dimerization. These interfaces are composed of two complementary surfaces that associate mainly by hydrophobic interactions. Similar interfaces have been identified in TEL, Polycomb and DGKδ1, and these SAM domains form extended polymers that have important regulatory roles. In the lipid kinase DGKδ1, SAM domain-mediated polymerization keeps the enzyme in an inactive form and localized to cytoplasmic vesicles. Upon EGF stimulation, DGKδ1 is recruited to the plasma membrane, where its substrate is located [25]. The SAM domain of the transcriptional repressors Polyhomeotic [24] and TEL [23] form helical polymeric filaments, suggesting a mechanism by which transcriptional repressor complexes might spread along the chromosome.

The amino-terminal 85 residues of Ack1 contain SAM domain-like sequences that are conserved across species [1]. Fusion of the Ack1 SAM domain sequence to GFP resulted in localization at the plasma membrane [1]. Deletion of the SAM domain from full-length Ack1 reduced the level of autophosphorylation in HeLa cells, suggesting the involvement of the N-terminus in enzyme regulation [1]. Here, we explore the role of the N-terminal domain in Ack1 localization and regulation. Our data suggest that the SAM domain promotes Ack1 dimerization at the plasma membrane to allow intermolecular autophosphorylation, analogous to the role of the transmembrane domains of receptor tyrosine kinases.

## Results

### Analysis of Ack1 autophosphorylation by Western blotting

To test for the importance of the amino-terminal SAM domain in Ack1 regulation, we created the deletion and truncation constructs that are shown in Figure 1A. In the construct ΔNt, the first 110 amino acid residues were deleted. NKD corresponds to the first 381 residues of Ack1 including the SAM domain and the kinase domain, and KD is the kinase domain alone.

We expressed these constructs in Cos7 cells and analyzed their activities in whole cell lysates by probing for phosphorylation of Y284. We previously identified Y284, which lies in the activation loop of Ack1, as the major autophosphorylation site [8]. We confirmed that Y284 is the major site in Cos7 cells, and that it represents a site of autophosphorylation rather than a site for another kinase (Additional file 1, Figure S1). Phosphorylated Ack1 was detected in the lanes corresponding to NKD and WT, but the lanes corresponding to KD and ΔNt did not show any detectable signal for pY284-Ack1 (Figure 1B, top panel). Thus, the level of Ack1 autophosphorylation was drastically reduced by the deletion of the SAM domain both in the context of the full-length protein (i.e., WT vs. ΔNt) and of the shorter constructs (NKD vs. KD). The membrane was reprobed with anti-HA antibody to determine total levels of the proteins in the blot (Figure 1B, middle panel). Additional examples are provided in Additional file 2, Figure S2. The ratio of pY284-Ack1 signal to total Ack1 signal for several blots is quantified in Figure 1C. These results suggest that the SAM domain plays an important role in the regulation of Ack1 by promoting its autophosphorylation.

We also analyzed the overall phosphotyrosine content of cells expressing the different Ack1 constructs (Figure 1B, bottom panel). Expression of full-length Ack1 resulted in the tyrosine phosphorylation of numerous cellular proteins, while fewer proteins were phosphorylated in ΔNt-expressing cells. For both full-length Ack1 and ΔNt, a band was present that co-migrated with the phosphorylated Ack1 protein, although the band was weaker for ΔNt. No signal was observed for ΔNt in the pY284 blot, but this construct contains other previously identified phosphorylation sites (Y826, Y857) that may account for the signal in the pTyr blot [28,29]. Expression of the NKD construct did not produce any drastic change in the pattern of phosphotyrosine-containing proteins as compared to untransfected Cos7 cells, but a tyrosine phosphorylated band migrating at the position of NKD was detected. In contrast, expression of the isolated kinase domain (KD) did not stimulate phosphorylation of cellular proteins compared to untransfected control cells, and no evidence for autophosphorylation was detected. This is consistent with the data in the pY284 blot, since this residue is probably the only one available for phosphorylation in KD and NKD. These data suggest that Ack1 N-terminus is important for its activation.

### The deletion of Ack1 N-terminus affects subcellular localization

**Figure 2:**
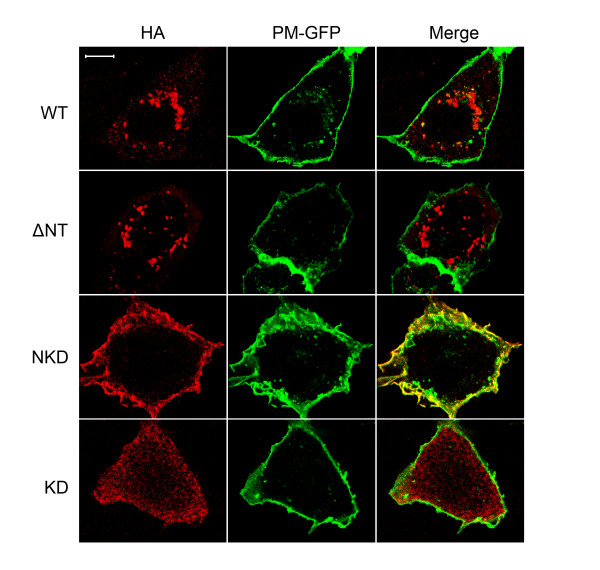
**Localization of Ack1 proteins**. Cos7 cells co-expressing wild-type or mutant HA-tagged Ack1 along with a membrane targeted form of GFP (PM-GFP) [40] were prepared for immunostaining and confocal microscopy as described in Materials and Methods. Ack1 proteins were detected with anti-HA antibodies. Anti-HA; left panels; PM-GFP, middle panels; merged images, right panels. Scale bar: 10 microns.

As reported earlier [30,31], WT Ack1 was localized to the cytoplasm in amorphous intracellular structures (Figure 2). ΔNT was present in similar large intracellular structures (Figure 2). By contrast, NKD was mostly localized to the plasma membrane, while the isolated kinase domain (KD) showed a diffuse cytosolic localization. The difference between NKD and KD localization patterns was consistent with the reported ability of the SAM domain to direct Ack1 to the plasma membrane [1]. The intracellular localization of WT Ack1 and ΔNT suggested that other regions of the protein may also contain targeting information, consistent with the observation that the C-terminus of Ack1 interacts with a number of proteins.

### Analysis of Ack1 autophosphorylation by quantitative immunofluorescence

**Figure 3:**
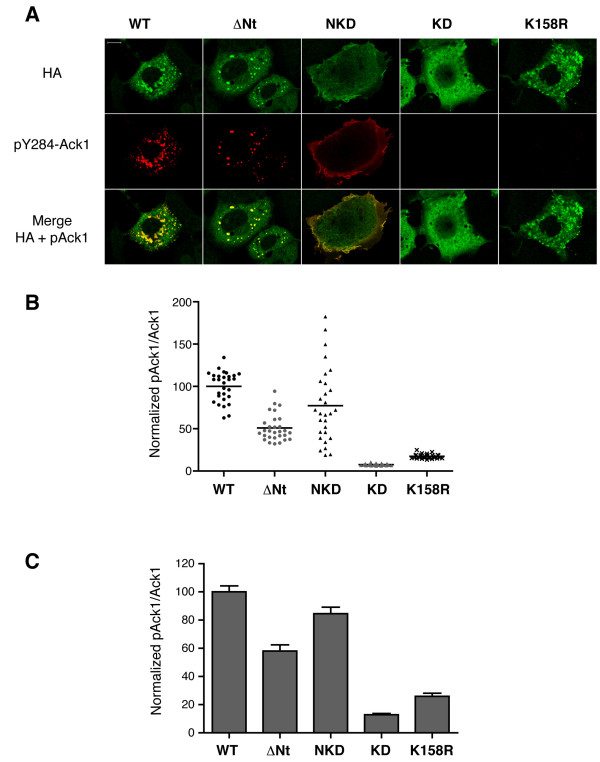
**Quantitative immunofluorescence analysis**. A, Cos7 cells expressing wild type or truncated forms of Ack1 were prepared for immunofluorescence using anti-phospho-Ack1 (pY284) and anti-HA antibodies. Top row: HA staining; middle row: pY284 staining; bottom row: merged images. The images in panel A were taken from the set used for quantitation and statistics in panels B and C. B, Immunofluorescence (anti-pY284 and anti-HA) was measured in three separate experiments (30 cells per experiment). The ratio of pY284Ack1/HA in each cell was calculated and normalized with respect to WT Ack1. Acquisition parameters were the same for all cells, and images shown as well as those used for quantitation were not further processed. A scatter plot for one representative experiment, with a line at the mean is shown. C, Bar graph showing the combined data of the three experiments. The data were compared using a one-way ANOVA test (P < 0.0001), followed by a Tukey's test of honest significant differences. All groups were significantly different from each other; P < 0.001 for all pairings, with the exception of KD vs. K158R (P < 0.01). Errors bars indicate standard error.

Next, we tested whether the various forms of Ack1 were active in all cellular sites. We studied the subcellular localization of the autophosphorylated, activated forms of Ack1 and the mutants by immunofluorescence analysis using anti-pY284 Ack1 antibody. In the cases where it was detected, the signal for pY284 Ack1 colocalized exactly with the distribution of total Ack1 (Figure 3A).

To determine the relative activities of the constructs, we quantitated anti-pY284 staining (for autophosphorylated Ack1) and anti-HA staining (for total Ack1) (Figures 3B and 3C). The ratios of fluorescence intensities (pY284/HA) were normalized to the value corresponding to wild type (WT = 100). Figure 3B shows results from a representative experiment, while 3C shows the merged data from three experiments. Consistent with the Western blotting results, the level of autophosphorylation was significantly decreased by the deletion of the N-terminus, both in the context of the full length and the minimal construct. The construct ΔNt showed a ~2-fold decrease in autophosphorylation compared to wild type, while the isolated kinase domain (KD) had a ~9-fold decrease in autophosphorylation compared to NKD. The autophosphorylation level of KD was not significantly different from the negative control (K158R, a kinase dead Ack1 mutant).

### Concentration-dependent activity of the purified Ack1 kinase domain

**Figure 4:**
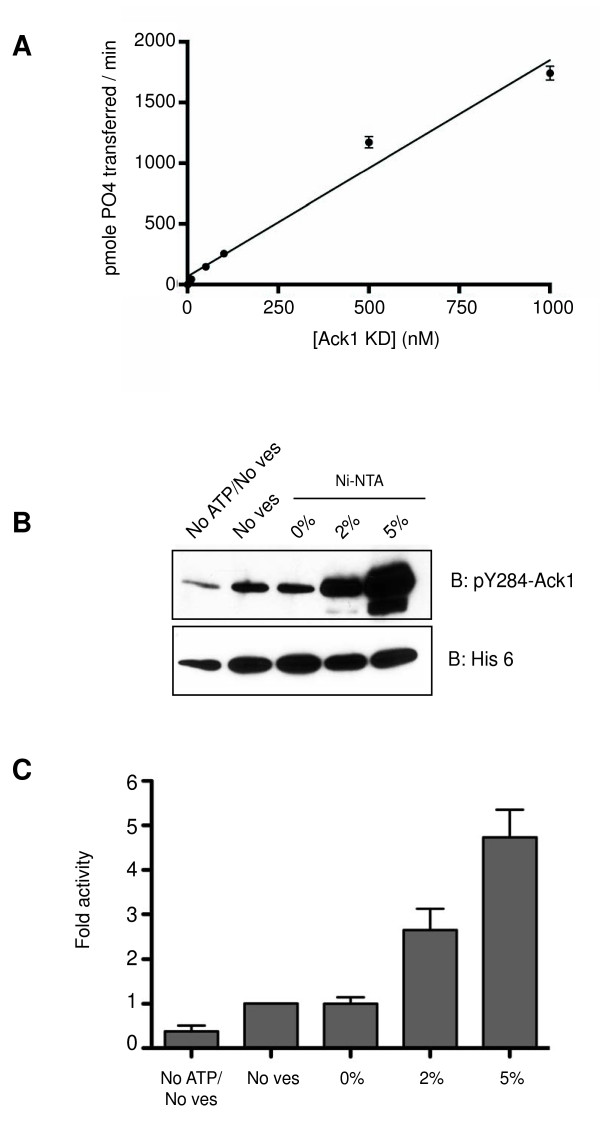
**Activity of purified Ack1 kinase domain**. A, purified Ack1 kinase domain is active *in vitro*. The catalytic activity of Ack1 kinase domain was measured towards a WASP-derived peptide at different enzyme concentrations. The figure is representative of three experiments. B, increasing local concentrations of purified Ack1 kinase domain at the surface of lipid vesicles stimulates autophosphorylation. The purified dephosphorylated Ack1 kinase domain was incubated in the presence or absence of ATP and in the presence of lipid vesicles containing different mole ratios of DOGS NTA-Ni. After the reaction, equal amounts of Ack1 kinase domain were separated by SDS-PAGE and analyzed by Western blotting using anti- phospho-Ack1 (pY284) and anti His_6 _antibodies. In these experiments, the enzyme concentration was 142 nM and the Ni-NTA was kept constant at 58.5 μM. In order to keep the Ni-NTA concentration constant, the amount of total lipids was varied inversely with the mole % of Ni-NTA in the vesicles. For 0% and 2% Ni-NTA, the total lipid concentration was 3.3 mM and for 5% Ni-NTA the lipid concentration was 1.3 mM. No ATP/No ves: reaction in the absence of vesicles and ATP. No ves: reaction in the absence of vesicles. The figure is representative of three experiments. C, quantitation of the western blots described in B. Densitometry readings were used to calculate the ratios of phosphorylated Ack1 (pY284 blot) to Ack1 (His_6 _blot). Error bars indicate standard error.

A possible explanation for the negligible autophosphorylation of the KD construct (Figures 1, 3) is that the isolated kinase domain might be inactive. Several tyrosine kinase catalytic domains (e.g., Csk, Fes) require the presence of accessory domains for activity [32,33]. To test this possibility, we introduced the Ack1 kinase domain into a recombinant baculovirus, expressed the protein in insect cells, and purified it to homogeneity. We tested the catalytic activity of Ack1 kinase domain by carrying out kinetic experiments using varying concentrations of a peptide substrate derived from the Tyr256 sequence of WASP (KVIYDFEKKKG). As shown in Figure 4A, the purified kinase domain was active towards the peptide. Experiments with varying ATP concentrations yielded a K_M_(ATP) value of 107 μM (data not shown). Thus, although the kinase domain expressed in cells is inactive, the isolated kinase domain has intrinsic tyrosine kinase activity. We hypothesized that the observed activity *in vitro *may be a function of the increased protein concentration under these conditions. The SAM domains in other proteins mediate polymerization processes that regulate activity [22], and the SAM domain of Ack1 acts as a membrane-targeting signal (Figure 2 and ref. [1]). In full-length Ack1, the SAM domain may increase the local concentration by causing dimerization or multimerization at the sites where it is recruited.

Kinases that dimerize and transphosphorylate show concentration-dependent activity. In order to test the effect of Ack1 concentration on autophosphorylation, we first dephosphorylated the His_6_-tagged enzyme purified from Sf9 cells using the *Yersinia *phosphatase Yop. Next, we used lipid vesicles to control the local concentration of Ack1 kinase domain. Dephosphorylated Ack1 was incubated with ATP in the presence of small unilamellar vesicles containing different mole fractions of a lipid with a Ni-NTA head group (DOGS-NTA-Ni). This method was used previously to demonstrate the activation of EGFR by formation of an asymmetric dimer [34]. Thus, in these experiments, we mimicked the dimerization function of the SAM domain by using the hexahistidine tag to control the local concentrations of the isolated kinase domain. The concentration of enzyme was kept constant, and the concentration of Ni-NTA was kept constant (by varying the total vesicle concentration) to ensure that any observed effects were due to changes in effective local concentration. Attachment of the purified kinase domain to vesicles caused a significant increase in autophosphorylation, as judged by anti-pY284 Western blotting (Figure 4B, C). The stimulation of autophosphorylation by DOGS-NTA-Ni was dose-dependent, as vesicles with 5 mole % DOGS-NTA-Ni produced a further increase over vesicles with 2 mole % DOGS-NTA-Ni. Vesicles that contained no Ni-NTA did not produce any increase in autophosphorylation compared to the activity in the absence of vesicles, indicating that that the observed effect was not due to any unspecific interaction between lipids and proteins (Figure 4B, C).

**Figure 5:**
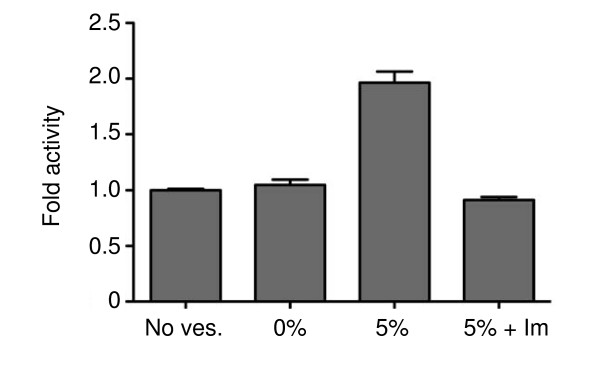
**Increasing local concentrations of purified Ack1 kinase domain at the surface of lipid vesicles increases catalytic activity**. The catalytic activity of Ack1 kinase domain towards a peptide was measured as in Figure 4. The activities of purified Ack1 kinase domain were measured in solution (no ves) or with vesicles containing 0 or 5 mole % of DOGS-NTA-Ni. In one experiment (5% + Im), the reaction was carried out in the presence of 500 mM imidazole. The enzyme concentration was 250-500 nM, peptide substrate was used at 1 mM, and the Ni-NTA concentration was kept constant at 30 μM. The figure shows the average results of nine experiments performed in triplicate. Errors bars indicate standard error.

The vesicles were also used to analyze the catalytic activity of purified Ack1 kinase domain towards a peptide substrate in a continuous spectrophotometric assay (Figure 5). The catalytic activity of the enzyme in the presence of vesicles containing 5 mole % DOGS-NTA-Ni was 2-fold higher than with vesicles containing no Ni-NTA, or than solution reactions (i.e., in the absence of vesicles). We confirmed that the effect was due to binding between the His_6_-Ack1 kinase domain and DOGS-NTA-Ni by performing the assay in the presence of 500 mM imidazole; under these conditions the Ack1 activity was comparable to the activity observed in the absence of vesicles or in solution. Taken together, the data show that artificially increasing the local concentration of the isolated kinase domain increases autophosphorylation and catalytic activity.

### Ack1 self associates in cells

**Figure 6:**
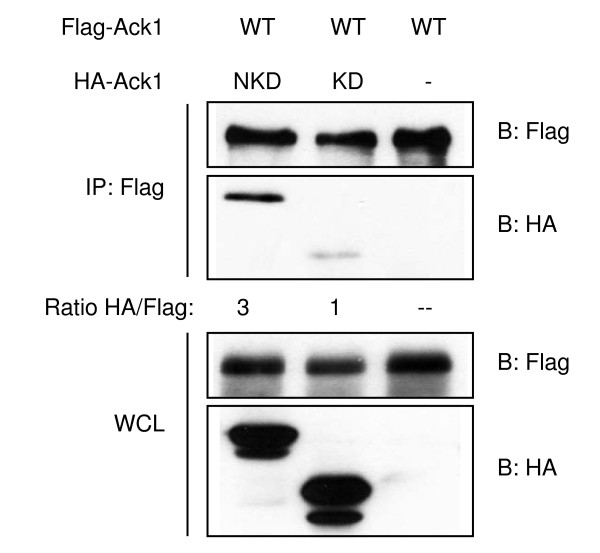
**Ack1 self-associates in cells**. Cos7 cells were transfected with Flag-tagged wild type Ack1 alone or together with HA-tagged Ack1 NKD or KD. Ack1 was immunoprecipitated from cell lysates with anti-Flag antibodies, and the immunocomplexes were analyzed by Western blotting with anti-Flag and anti-HA antibodies. Densitometry readings of the immunoprecipitated proteins (top panels) were used to calculate the ratios of immunoprecipitated HA-Ack1 to immunoprecipitated Flag-Ack1. The HA:Flag ratio for KD was arbitrarily set to 1. Samples of whole cell lysates were analyzed for the presence of the transfected proteins (bottom panels). The figure is representative of three experiments.

We investigated the capacity of Ack1 constructs to self-associate in cells. Cos 7 cells were transfected with Flag-tagged full length Ack1 alone or together with HA-tagged NKD or KD. Flag-Ack1 was immunoprecipitated from the cell lysates using anti-Flag antibody, processed for SDS-PAGE and transferred to PVDF membranes. Blots were probed for the presence of co-immunoprecipitated HA-tagged proteins. The densitometry readings corresponding to Flag-tagged Ack1 and to HA-tagged Ack1 in the immunocomplexes were used to calculate the ratio HA-Ack1/Flag-Ack1. We found that HA-NKD coimmunoprecipitated with Flag Ack1 3-fold more strongly than HA-KD (Figure 6). Thus, although there is residual self-association between the kinase domain and full-length Ack1, these data suggest that the N-terminal region strengthens the association.

## Discussion

SAM domains are a diverse group of protein-protein interaction motifs that frequently mediate homo- or hetero- dimerization and oligomerization. In several enzymes, SAM domains regulate activity by localized polymerization at the membrane or in the nucleus. In this work, we show that the SAM domain of Ack1 is required for autophosphorylation. Our data suggest that the N-terminus of Ack1 mediates self-association, and that the increased local concentrations result in increased autophosphorylation and kinase activity.

To study the role of the Ack1 N-terminus in autophosphorylation, we deleted the first 110 amino acids of the polypeptide (ΔNt) and we compared it to the full-length protein (WT). In addition, we created a minimal construct that contains only the N-terminus and the kinase domain (NKD) and we compared it to the isolated kinase domain (KD). Since Ack1 is a large protein that contains several different regions, some of whose roles are not completely understood, this simplified system proved to be useful to dissect the role of the N-terminus in Ack1 activation and intracellular localization.

The deletion of the Ack1 N-terminus produced a significant decrease in Ack1 autophosphorylation in cells. Similar trends were observed by two different methods, although the Western blot quantitation method appears to have a broader dynamic range than the quantitative immunofluorescence. Autophosphorylation of ΔNt was 15-fold lower than WT Ack1 by Western blotting (Figure 1) and 2-fold lower by immunofluorescence (Figure 3). The effect on the minimal NKD construct was more pronounced: autophosphorylation of KD was 27-fold lower than NKD by Western blotting and 9-fold lower by immunofluorescence. In the immunofluorescence assays (Figure 3), autophosphorylation of KD was significantly lower than ΔNt. A possible explanation for this difference is that other regions of Ack1, in addition to the N-terminus, may mediate interactions or provide inputs that result in increased autophosphorylation.

Consistent with published data [1], we observed that Ack1 N-terminus is a membrane-targeting domain (Figures 2 and 3). The minimal constructs NKD and KD show very distinct intracellular localizations: NKD is clearly localized at the plasma membrane and phosphorylated, while KD is diffuse and cytosolic, and unphosphorylated at Y284 (Figures 2 and 3). The situation is more complex in the cases of WT and ΔNt. They both show a punctate distribution that is not identical to either NKD or KD. This suggests that the localization of full-length Ack1 results from the cumulative effect of interactions mediated by the SAM domain and other regions of the protein. It is likely that interactions between full-length Ack1 and other proteins including clathrin [2], sorting nexin 9 [7], and ubiquitinated EGFR [4] play a role in determining subcellular localization.

In the context of full-length Ack1, the primary function of the SAM domain may be to promote enzyme activation. We used an *in vitro *system to explicitly test the hypothesis that self-association is an activation mechanism for Ack1. We found that Ack1 kinase domain attached to vesicles showed a concentration-dependent increase in autophosphorylation and in phosphorylation of an exogenous substrate (Figures 4 and 5). FLAG-Ack1 co-immunoprecipitated more weakly with HA-KD than with HA-NKD, suggesting that the N-terminus promotes Ack1 homodimerization (Figure 6). These results suggest that increasing the local concentration of Ack1 catalytic domain by SAM domain-mediated dimerization is potentially a mechanism for enzyme activation.

Counterbalancing mechanisms presumably exist to reverse the SAM domain-mediated activation of Ack1. Although these mechanisms are undefined at present, the domain arrangement of Ack1 is complex, and it is likely that other regions of the polypeptide are involved in Ack1 downregulation. We have shown that the Mig6-homology region (MHR) located at the C-terminus of Ack1 is involved in an intramolecular autoinhibitory interaction [31]. The MHR is also capable of binding to EGFR kinase domain *in vitro *(V.P.E., W.T.M.; unpublished observations) and in cell lysates [4]. This Ack1-EGFR interaction could potentially be involved in the dynamic regulation of Ack1 activity. Growth factor treatment of cells promotes phosphorylation of the Ack1 C-terminus [29,30], which could potentially destabilize autoinhibitory interactions and unmask the SAM domain for membrane recruitment and Ack1 activation.

## Conclusions

Our data suggest that: (1) The N-terminal SAM domain of Ack1 is required for full Ack1 autophosphorylation and kinase activity; (2) The SAM domain has the potential to drive Ack1 to the plasma membrane, although in the context of full-length Ack1 other protein-protein interactions make important contributions; (3) Increasing the local concentration of Ack1 stimulates *in vitro *kinase activity; and (4) Ack1 forms dimers (or higher-order multimers) in cells.

## Methods

### Reagents and antibodies

Bovine serum albumin, leupeptin, aprotinin, PMSF, sodium vanadate, sodium fluoride, pyruvate kinase/lactate dehydrogenase enzymes, and EZview red anti-FLAG M2 affinity gel were obtained from Sigma. Egg PC and DOGS-NTA-Ni were from Avanti Polar lipids, Inc. Trypsin-EDTA solution was from Mediatech Inc. Primary antibodies were obtained from the following companies: rabbit polyclonal anti-Ack1, rabbit polyclonal anti-pY284 Ack1 and mouse monoclonal anti-phosphotyrosine clone 4G10 were from Millipore, mouse monoclonal anti-His_6 _was from Covance, rat monoclonal anti-HA high affinity clone 3F10 was from Roche. The primary antibodies used for immunofluorescence were mouse monoclonal anti-HA from Applied Biological Materials and rabbit anti-HA tag antibodies from Sigma-Aldrich. The secondary antibodies were horseradish peroxidase linked secondary antibodies (donkey anti-rabbit IgG and sheep anti- mouse IgG horseradish) from GE Healthcare. Alexa Fluor (AF)-594-transferrin and AF-488- and AF-594-goat anti-mouse and goat anti-rabbit IgG secondary antibodies were from Molecular Probes, Invitrogen. The plasmid PM-GFP, encoding the NH_2_-terminal 10-amino acid acylation sequence of Lyn linked to GFP, was provided by Pietro De Camilli (Howard Hughes Medical Institute, Yale University School of Medicine).

### Cell culture

Mammalian cells were maintained in DMEM (Cellgro, Mediatech, Inc) supplemented with 10% fetal bovine serum (Sigma) and 1000 IU/ml penicillin, 1000 IU/ml streptomycin, 25 ng/ml amphotericin B (Cellgro, Mediatech, Inc). The Sf9 insect cells were maintained in Sf-900 medium (Gibco) supplemented with 5% fetal bovine serum and 1000 IU/ml streptomycin, 25 ng/ml amphotericin B (Cellgro, Mediatech, Inc).

### Cloning and site-directed mutagenesis

Plasmid pXJ-HA-Ack1 encoding full length Ack1 was a kind gift from Dr. Edward Manser (Institute of Molecular and Cell Biology, Singapore). A plasmid encoding EGFP-clathrin light chain A in pEGFP-C3 [35] was the gift of Lois Greene (National Heart, Lung, and Blood Institute, National Institutes of Health, Bethesda, MD). The plasmid pXJ-HA-Ack1 was modified to produce the different constructs used in this study. The ΔNt construct was generated by PCR amplification of DNA encoding residues 330-1085 using primers that contained the restriction sites BamHI and HindIII. To generate the NKD construct, a termination signal (TGA) was inserted after codon 385 by site-directed mutagenesis. The KD construct was made by subcloning the region encoding Ack1 kinase domain (residues 110 to 385) into pXJ-HA. To produce FLAG-tagged Ack1, the coding region region for full length Ack1 was subcloned from pXJ-HA into the plasmid p(3X)FLAG CMV 7.1 (Sigma) using the restriction sites BamHI and HindIII.

### Cell transfection and Western blotting

For Western blotting, Cos7 cells (from American Type Culture Collection (ATCC), Manassas, VA) (3 × 10^6^) were plated in 15 cm diameter dishes. After 24 hours, the cells were transfected using 30-60 μg DNA with TransIT reagent (Mirus) at a ratio of 2 μl TransIT per μg of DNA. After 24 hours, the reagent was removed, and the cells were cultured for an additional 24 hours. The cells were harvested, washed twice in PBS, and lysed using RIPA buffer (50 mM Tris-HCl pH 7.4, 150 mM NaCl, 5 mM EDTA, 1% sodium deoxycholate, 1% Nonidet P-40) supplemented with the protease inhibitors leupeptin (10 μg/ml), aprotinin (10 μg/ml), PMSF (200 μM) and the phosphatase inhibitors Na_3_VO_4 _(0.2 mM) and NaF (10 mM). Lysates (30 μg) were separated by 10% SDS-PAGE, transferred to PVDF membrane, and probed with appropriate antibodies.

For immunofluorescence, the transfections were carried out as follows: Cos7 cells were maintained in Dulbecco's modified Eagle's medium with 10% iron-supplemented calf serum (JRH, Lenexa, KS) and penicillin/streptomycin. Transient transfection of cells seeded on acid-washed glass coverslips was performed using fully-deacylated polyethylenimine (PEI) reagent, prepared from 200 kDa poly(2-ethyl-2-oxazoline) (Sigma-Aldrich, St. Louis, MO) as described [36]. Transfection mixtures contained 2 μg DNA and 12 μl PEI reagent per 35 mm dish. Cells were examined one day after transfection.

### Fluorescence microscopy

Cells grown on glass coverslips were fixed in phosphate-buffered saline (PBS; 150 mM NaCl, 20 mM phosphate buffer, pH 7.4) containing 3% paraformaldehyde for 30 minutes, permeabilized with PBS containing 0.5% Triton X-100 and blocked with PBS containing 3% bovine serum albumin (BSA) and 10 mM glycine for 1 hour. Cells were incubated with primary antibodies for 1 hour and secondary antibodies for 30 minutes, both in PBS with 3% BSA and 10 mM glycine. Cells were photographed and images were captured using a Zeiss LSM 510 META NLO laser scanning confocal microscope using a 100x oil immersion objective. For quantification, a region of interest (ROI) was drawn around individual cells, and the histogram macro on the Zeiss LSM software was used to quantify the mean fluorescence intensity of each cell in both red and green channels, and the ratio of anti-pY284 to anti-HA staining in each cell was calculated. Acquisition parameters were the same for all cells, and images shown as well as those used for quantitation were not further processed. 30 cells expressing each construct were analyzed in each of three separate experiments.

### Protein expression and purification

A recombinant baculovirus encoding the Ack1 kinase domain (residues 110-385; Prieto-Echagüe et al. [31]) was used to infect 400 ml of Sf9 cells at 2 × 10^6 ^cell/ml with MOI~5. The infected cells were collected after 48 hours and lysed in a French pressure cell in 20 mM Tris-HCl buffer (pH 8.0), containing 5 mM 2-mercaptoethanol, 10 μg/ml leupeptin, 10 μg/ml aprotinin, 1 mM PMSF, and 1 mM Na_3_VO_4_. The cell homogenates were cleared by centrifugation at 40,000 g for 30 min, filtered using a 0.8 um filter, and applied to a 4 ml Ni-NTA column (Qiagen). First, the column was washed with 120 ml of 20 mM Tris-HCl buffer (pH 8.0) containing 2 mM imidazole, 0.5 M NaCl, 10% glycerol, 5 mM 2-mercaptoethanol, 2 mM Na_3_VO_4_. The second wash was with 40 ml of 20 mM Tris-HCl buffer (pH 8.0) containing 1 M NaCl and a third wash was carried out with 40 ml of 20 mM Tris-HCl buffer (pH 8.0). Buffer containing 20 mM Tris-HCl (pH 8.0),100 mM imidazole, 5 mM 2-mercaptoethanol and 10% glycerol was used to elute the His_6_-tagged proteins. The fractions containing Ack1 kinase domain were pooled, supplemented with 20% glycerol, and stored at -80°C.

### Co-immunoprecipitation studies

Cos 7 cells were transfected with full length FLAG-Ack1 alone or cotransfected with HA-tagged Ack1 (NKD or KD) and lysed after 48 hours using RIPA buffer, as described for the Western blotting experiments. Cell lysates (450-750 μg) were incubated overnight at 4°C with 40 μl of FLAG M2 affinity gel. The immunocomplexes were washed 5 times with 1 ml RIPA buffer, eluted from the beads using 35 μl of 2X Laemmli buffer and separated by SDS-PAGE. The proteins were transferred to PVDF membranes and analyzed by immunoblotting using anti-HA antibody and anti-FLAG antibody.

### Preparation of small unilamellar vesicles

The lipids egg PC and DOGS-NTA-Ni were mixed in different molar ratios in glass tubes. The chloroform from 500 μl of the mixture was evaporated under an argon stream to form a thin film, and the lipids were dried under vacuum for at least one hour. The dried lipids were resuspended to create large multilamellar vesicles by the addition of 500 μl of rehydration buffer (20 mM Tris pH 7.4 and 10 mM MgCl_2_) followed by 4 cycles of freezing, thawing and 30 seconds vigorous vortexing. Small unilamellar vesicles were produced by passing the large multilamellar vesicles through a polycarbonate filter (pore size: 100 nm) 10 times, using a mini-extruder (Avanti Polar lipids, Inc). The vesicles were used for *in vitro *assays on the same day of preparation.

### In vitro autophosphorylation reactions

Purified His_6_-Ack1 kinase domain was dephosphorylated by incubating 10 μg of protein with immobilized GST-tagged *Yersinia *phosphatase (Yop) for 30 minutes at room temperature. For the autophosphorylation reactions on vesicles, dephosphorylated Ack1 (500 ng) was incubated with kinase buffer (100 mM Tris-HCl pH 7.4, 10 mM MgCl_2_, 25 mM vanadate, and 500 mM ATP) in the presence of vesicles containing different mole fractions of DOGS-NTA-Ni (0%, 2% or 5%). Reactions proceeded for 15 minutes at room temperature and were stopped by adding Laemmli buffer and boiling for 5 minutes. Samples were analyzed by SDS-PAGE. The proteins were transferred to PVDF membranes and analyzed by immunoblotting using anti-phosphoAck1 (Y184) and anti-His6 antibodies.

### In vitro kinase assay in solution and with vesicles

The catalytic activity of the purified Ack1 kinase domain towards a peptide substrate was measured using a continuous spectrophotometric assay [37,38]. Reactions were performed at 30°C in a final volume of 50 μl. The substrate used was a peptide containing the tyrosine phosphorylation site in the protein WASP that is phosphorylated by Ack1 [39]. The reactions contained 100 mM Tris pH 7.4, 10 mM MgCl_2_, 2 mM ATP, 1.5 mM phosphoenolpyruvate (PEP), 90 units/ml pyruvate kinase, 109 units/ml lactate dehydrogenase, and 1.2 mg/ml NADH. For determination of kinetic constants, the peptide substrate concentration was varied from 5 μM to 5 mM; for experiments with vesicles, the peptide concentration was 625 μM.

## Abbreviations

CAS: Crk-associated substrate; DACK: *Drosophila melanogaster *Ack; E3: ubiquitin-protein isopeptide ligase; EGF: epidermal growth factor; EGFR: EGF receptor; GFP: Green fluorescent protein; Grb2: Growth factor receptor-bound protein 2; HA: Hemagglutinin; HCK: Hemopoietic cell kinase; NRTK: nonreceptor tyrosine kinase; PBS: Phosphate-buffered saline; PMSF: phenylmethylsulfonyl fluoride; PVDF: Polyninylidene difluoride; SAM: sterile alpha motif; SH: Src homology; SNX9: sortin nexin 9; TEL: translocation Ets leukemia; Tnk1: thirty-eight-negative kinase 1; WASP: Wiskott-Aldrich syndrome protein; WT: wild type; YOP: Yersinia outer protein.

## Authors' contributions

VPE carried out the molecular cloning and mutagenesis, cell transfections, Western blotting, protein expression and purification, co-immunoprecipitation, preparation of unilamellar vesicles, autophosphorylation and kinase assays, performed the statistical analysis of the quantitative immunofluorescence data, and drafted the manuscript. AG carried out the immunofluorescence studies. DAB participated in the design of the study and helped to draft the manuscript. WTM conceived of the study, and participated in its design and coordination and drafted the manuscript. All authors read and approved the final manuscript.

## Supplementary Material

Additional file 1 **Fig S1**. Supplementary data showing tyrosine 284 is the major autophosphorylation site. Click here for file

Additional file 2 **Fig S2**. Supplementary data showing N-terminus is required for Ack1 autophosphorylation. Click here for file
