# Supplementary material for: Regulation of Ack1 localization and activity by the amino-terminal SAM domain
Source: BMC Biochem. 2010 Oct 27;11:42. doi: 10.1186/1471-2091-11-42 (PMC2987765; doi:10.1186/1471-2091-11-42)
Supplement: Additional file 1 — Fig S1. Supplementary data showing tyrosine 284 is the major autophosphorylation site. [file 1471-2091-11-42-S1.PDF]

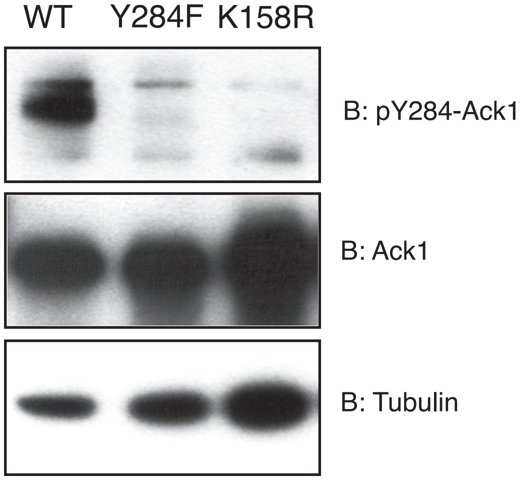

Figure S1: Tyrosine 284 is an autophosphorylation site. Wild-type Ack1, Y284F (autophosphorylation site) or K158R (kinase dead) mutants of Ack1 were expressed in Cos7 cells. Cell lysates were probed with anti-phospho Ack1 (pY284), anti-Ack1 and anti-tubulin antibodies, as indicated.
