# Supplementary material for: Regulation of Ack1 localization and activity by the amino-terminal SAM domain
Source: BMC Biochem. 2010 Oct 27;11:42. doi: 10.1186/1471-2091-11-42 (PMC2987765; doi:10.1186/1471-2091-11-42)
Supplement: Additional file 2 — Fig S2. Supplementary data showing N-terminus is required for Ack1 autophosphorylation. [file 1471-2091-11-42-S2.PDF]

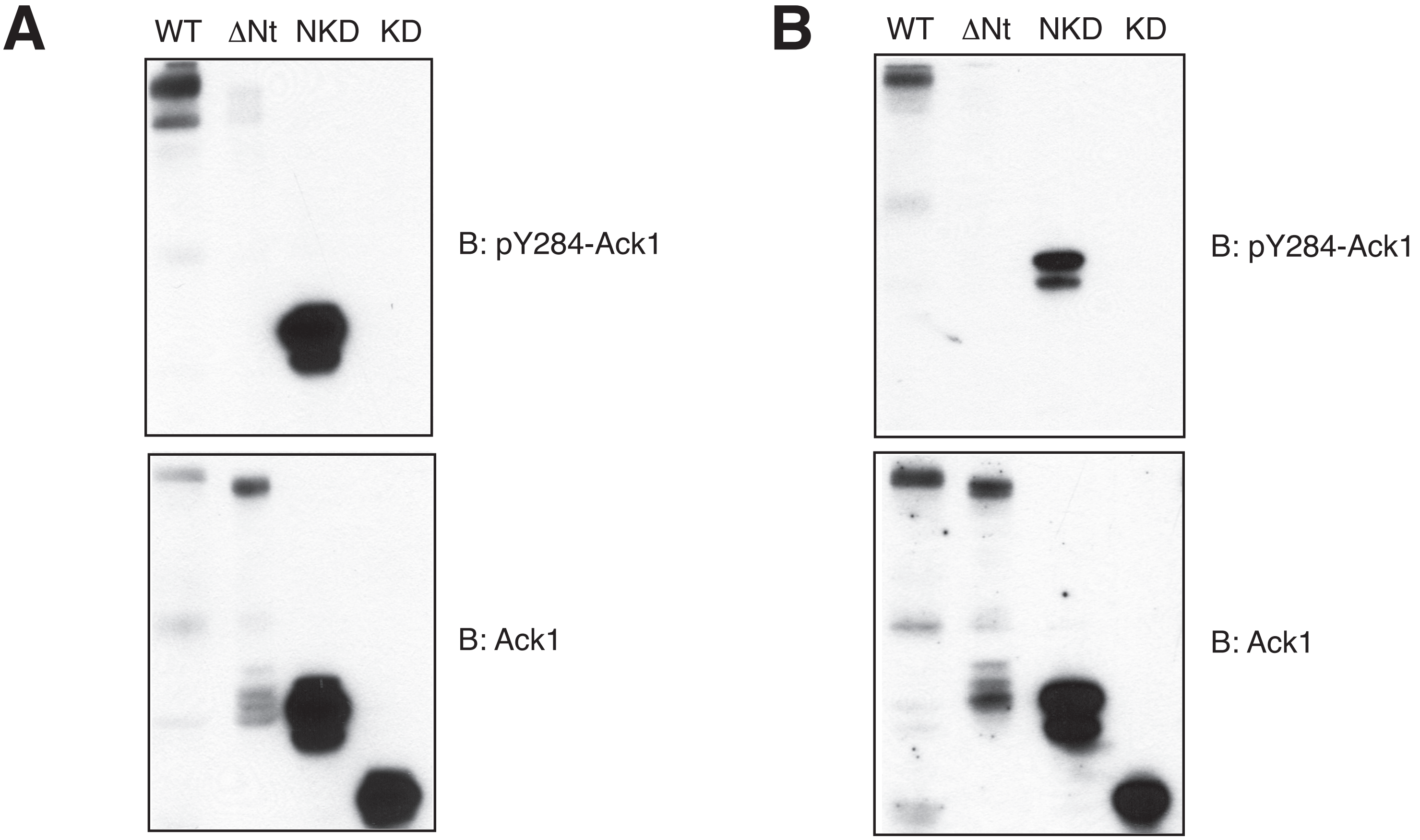

Figure S2. The N-terminus of Ack1 is required for autophosphorylation in cells. Lysates from Cos7 cells expressing wild type or truncated forms of Ack1 were probed with anti- phospho Ack1 (pY284), anti-HA, and anti-phosphotyrosine antibodies as indicated. The figure shows additional replicates of the data in Fig. 1B.
